# Hearing Efficiency in Oral Submucous Fibrosis: A Clinical Study

**DOI:** 10.1007/s12070-020-02246-5

**Published:** 2020-10-29

**Authors:** J. S. Shah, Nutan Lunagariya

**Affiliations:** Oral Medicine and Radiology, Govt. Dental College and Hospital, Ahmedabad, Gujarat India

**Keywords:** Palatal, Paratubal muscles, Eustachian tube, Audiometry

## Abstract

Oral Submucous fibrosis (OSMF) is a chronic insidious disease of oral mucosa that occurs due to areca-nut chewing, consumption of chillies, autoimmunity and genetic predisposition. The disease starts with burning sensation and inability to tolerate spicy foods with gradual reduction in mouth opening due to fibrosis of the oral mucosa. The extension of fibrosis into the naso pharynx leads to reduction in hearing efficiency. As very few studies had been done to evaluate the hearing disability in OSMF patients, this study had been undertaken to prove the same. To evaluate hearing efficiency in patients with Oral Submucous Fibrosis of various grades of severity. Presentation includes 30 patients of osmf with various grades and evaluated for hearing efficacy by audiometry. Hearing threshold was compared in different grades of osmf. The present study revealed a significant association between OSMF and hearing deficit. Involvement of the palatal muscles with OSMF may decrease the patency of the Eustachian tube, leading to conductive hearing loss. Therefore, the protocol for managing OSMF patients should include ENT consultation and treatment for hearing deficit in order to increase the success rate of treatment.

## Introduction

Oral submucous fibrosis (OSMF) is a chronic insidious disease, affecting any part of the oral cavity and sometimes the pharynx. Although occasionally preceded by and/or associated with vesicle formation, it is always associated with a juxta-epithelial inflammatory reaction followed by fibroelastic changes of the lamina propria, with epithelial atrophy leading to stiffness of the oral mucosa and causing trismus and inability to eat [1, 2].

Symptoms of this disease include burning sensation of the oral mucosa, ulceration and pain, reduced movement and depapillation of tongue, blanching and leathery texture of oral mucosa, loss of pigmentation of oral mucosa, and progressive reduction of mouth opening. Advanced cases show signs of loss of hearing due to blockage of eustachian tubes and difficulty swallowing because of esophageal fibrosis [1].

Amongst structures communicating with the oral cavity the eustachian tube (pharyngotympanic tube) connects the middle ear cavity with the nasopharynx. Opening and closing functions of the eustachian tube are physiologically important [1]. Normal opening of the eustachian tube equalizes atmospheric pressure in the middle ear; closing of the eustachian tube protects the middle ear from unwanted pressure fluctuations and loud sounds. Abnormal or impaired eustachian tube functions (i.e., impaired opening or closing) may cause pathological changes in the middle ear. This in turn can lead to hearing disabilities.1 In OSMF, there can be failure of eustachian tube to effectively regulate air pressure. As eustachian tube function worsens, air pressure of middle ear falls and ear sounds are perceived as muffled and may cause impaired hearing [1, 3].

However there is a paucity of information related to the involvement by fibrosis of areas adjoining the oral cavity eg. Ear (Eustachian tube), Oro-pharynx, Pharynx and very few studies available correlating the eustachian tube dysfunction with various clinical stages of OSMF and its association with increase or decrease in the severity of the disease process. Therefore, this study was designed to evaluate eustachian tube function in OSMF patients and to correlate it with various clinical stages of the disease which may be helpful in assessing the morbidity and in identifying the overall prognosis to find more appropriate therapeutic interventions [3, 4].

## Material and Methods

The present study was carried out in the Department of Oral medicine and radiology, Government Dental College and Hospital Ahmedabad on 30 patients of OSMF after ethical approval by the Institutional Ethical Committee. After obtaining written informed consent, the clinical data of the patients was obtained by taking thorough case history and clinical examination. After the clinical examination, the diagnosis of OSMF was made on the basis of history and characteristic clinical features of the disease. Depending on the clinical findings, cases were divided into grade I, II and III. (Early, Moderate, Severe grade.)

### Clinical Criteria for Grading of OSMF

#### Grade I (Early OSMF)

Burning sensation on hot and spicy food, blanching, palpable fibrosis of buccal mucosa and fibrosis of faucial pillars, pterygomandibular raphae and soft palate; Mouth opening 25–35 mm.

#### Grade II (Moderate OSMF)

Burning sensation on hot and spicy food; Palpable fibrosis of buccal mucosa, faucial pillars, pterygomandibular raphae and soft palate extends anteriorly to involve labial mucosa, floor of the mouth and tongue; Tongue movements are restricted to some extents; Loss of flexibility of buccal mucosa; Mouth opening 15 mm–25 mm.

#### Grade III (Severe OSMF)

Burning sensation on absence of stimuli; Severe fibrosis of entire oral cavity; Severe restriction of tongue movements; Severe loss of flexibility of buccal mucosa; Circular band (fibrotic rim) around lips and mouth; Fibrosis of soft palate and shrunken uvula; Difficulty in swallowing and deglutition; Difficulty in speech and nasal voice; Restricted mouth opening less than 15 mm.

**Fig. 1:**
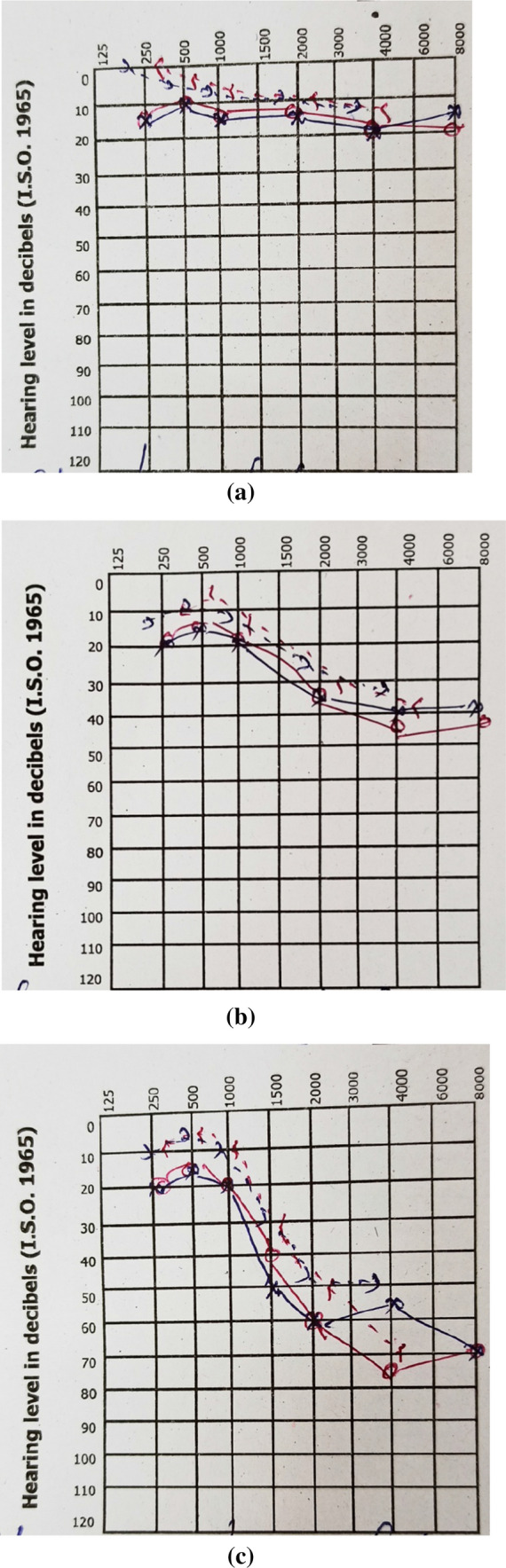
**a** Plotted graph show normal hearing level in 10–15 dB, **b** Plotted graph show Mild hearing level in 26–40 dB, **c** Plotted graph show Moderate to Severe hearing loss in 41–70 dB

Examination of the ear was done in ENT Department and they were ruled out other causes of hearing loss such as tympanic membrane perforation, cholesteatoma, previous surgery and ear infections. Pure tone audiometry (PTA) was performed in all selected cases. Both the right and left ears (total 60 ears) were evaluated for hearing loss. Hearing level in decibel above the normal threshold is plotted. (Fig. 1a, b, c) The frequency tested usually ranged from 125 to 8000 Hz. The amount of intensity that has to be raised above the normal level is the measure of degree of the hearing impairment at that frequency. Depending upon the AC–BC Gap values, hearing impairment was quantitatively graded into several categories as follows: [1, 4, 5]

1. 10–15 dB—Normal Hearing.
2. 16–25 dB—Minimal Hearing Loss.
3. 26–40 dB—Mild.
4. 41–55 dB—Moderate.
5. 56–70 dB—Moderate to Severe.
6. 71–90 dB—Severe.
7. Above 90 dB is profound deafness.

All subjects were evaluated for hearing loss according to age, and grades of OSMF and it tabulated as shown below.

## Result and Discussion

OSMF is predominantly a disease of the oral cavity and oropharynx. The disease is characterized by progressive fibrosis involving the mucus membrane of the mouth, mainly the buccal mucosa, soft palate, lip mucosa, and anterior pillars. It rarely affects the membrane lining of the pharyngeal box or vocal cords, but is capable of involving the eustachian tube [2, 5–7]. It affects about 0.2–1.2% of Indian population with predominantly seen in people of South Asia and South–East Asia – India, Bangladesh, Sri Lanka, Pakistan, Taiwan, Southern China, etc. where consumption of arecanut or its flavoured formulations or as an ingredient in the betel quid is more prevalent. [8, 9]. The malignant transformation rate of oral submucous fibrosis has been found to be 4%–13% worldwide and 7.6% in Indian population [5, 10, 11].

OSMF mainly affects in the second and third decades of life having male predominance [2, 5, 7]. Table 1 shows correlation of grade of OSMF with loss of hearing according to age group. In the present study we observed the subjects ranged in the age of 16–60 years. In Grade I OSMF, majority of the subjects were having age from 15 to 30 years. In Grade II OSMF, majority of the subjects were having age from 31 to 45 years. In Grade III OSMF, majority of the subjects were having age from 31 to 45 and 46 to 60 years. Statistically, significant difference was present between age groups and OSMF Grades. (*P* ≤ 0.005) Most of these patients were in the second and third decade of life [83.33%] patients being in the age group of 15–45 years. This is in accordance with the studies by Gupta et al. and Shah et al. [6, 7].

**Table 1:** Correlation of grade of OSMF with loss of hearing according to age group

Age (In Year)	Grade I (Total = 5) [16.67%]		Grade II (Total = 13) [43.33%]		Grade III (Total = 12) [40%]	
	Normal (Total = 5)[100%]	Loss of Hearing (Total = 0)[0%]	Normal (Total = 8)[61.53%]	Loss of Hearing (Total = 5) [38.46%]	Normal (Total = 7) [58.33%]	Loss of Hearing (Total = 5) [41.67%]
15–30 (Total = 11)	4[80%]	0	3[37.5%]	2[40%]	2[28.57%]	0
31–45 (Total = 14)	1[20%]	0	5[62.5%]	3[60%]	3[42.85%]	2[40%]
46–60 (Total = 5)	0[0%]	0	0[0%]	0	2[28.57%]	3[60%]

*P* Value ≤ 0.05 Significant

(Level of Significance *P* ≤ 0.05, Pearson Chi Square test)

Table 2 shows categories of hearing loss association with grades of OSMF. In the present study, pure tone audiometry of 60 ears in 30 OSMF patients revealed that hearing was normal in 42 ears (70%), minimal hearing loss was present in 5 ears (8.33%), mild hearing loss was present in 6 ears (10%) and moderate hearing loss was evident in 7 ears (11.66%). These results were in accordance with the study conducted by Gupta et al. (10), where hearing was found to be normal in 79.2% of ears, mild to moderate hearing loss was evident in 18.0%, and hearing loss was evident in 2.8%. Shah et al. reported that out of 54 ears in their OSMF group, hearing was normal in 67%, mild hearing loss was found in 22%, and moderate mixed hearing loss was present in 11%.Statistically, no significant difference was present between OSMF Grades and grade of hearing loss. (*P* > 0.05).

**Table 2:** Categories of hearing loss association with grades of OSMF

OSMF Grade (Total Ears = 60) [100%]	Normal H (Total = 42) [70%]	Minimal HL (Total = 5) [8.33%]	Mild HL (Total = 6) [10%]	Moderate HL (Total = 7) [11.66%]
Early OSMF	10 [23.80%]	0	0	0
Moderate OSMF	17[40.47%]	4[80%]	2[33.33%]	3[42.85%]
Severe OSMF	15[35.71%]	1[20%]	4[66.67%]	4[57.14%]

*P* Value ≤ 0.05 Significant

(Level of Significance *P* ≤ 0.05, Pearson Chi Square test)

In the present study there was significant difference noticed in hearing loss for group 1 and group 3 (early OSMF and severe OSMF) Group 3 was significantly associated with moderate hearing loss as compared with any other group in both the right and left ears. These results were in accordance with those of Chaudhary et al. Dysfunction of the eustachian tube in OSMF may be due to fibrosis of the palatal muscles.

**Fig. 2:**
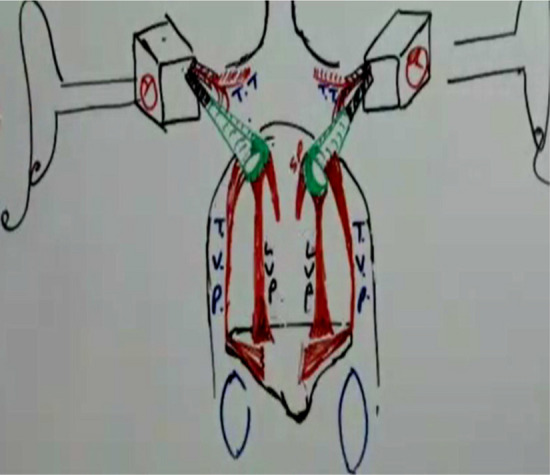
Schematic diagram of relationship of eustachian tube and palatal muscles (levator veli palatine (LVP), tensor veli palatini, (TVP) tensor tympani (TT) and salpingopharyngeus (SP)

Palatal involvement is seen in more than 50% of patients with fibrosis evident in the faucial pillars. Involvement of the palatal and paratubal muscles (levator veli palatini, tensor veli palatini, tensor tympani and salpingopharyngeus), which regulate the patency and function of the pharyngeal orifice. (Fig. 2) In OSMF there is further narrowing of the normally small opening of the pharyngeal orifice of the eustachian tube, results in failure to effectively regulate air pressure. Leading to pain in the ear along with loss of hearing [1, 4, 7]. Thus, hearing loss can be correlated with the degree of fibrosis of the palatal muscles, thus reducing the patency of the eustachian tube. As fibrosis of the oropharynx leads to altered perception of sound, it is evident in the advanced stages of OSMF because of alterations in the patency of the Eustachian tube, which may occur due to progressive fibrosis of the palatal muscles, being directly proportional to the stage of the disease.

Table 3 shows category of hearing loss according to age group. Age group of 15–30 years shows hearing loss in 3 ear, in which was minimal hearing loss in 2 ears and Moderate in 1 ear. Age group of 46–60 years shows moderate hearing loss in 6 ears. Out of 6, in 4 ear moderate hearing loss and in 2 mild hearing loss. Statistically, no significant difference was present between Age Groups and types of hearing loss. (*P* > 0.05. Hearing efficiency reduces as age advances. Therefore it might be a compounding factor too.

**Table 3:** Shows category of hearing loss according to age group

Age (In Year) (Total Ears = 18)	Minimum Hearing Loss (Total = 5)		Mild Hearing Loss (Total = 6)		Moderate Hearing Loss (Total = 7)	
	Right Ear (Total = 2) [40%]	Left Ear (Total = 3) [60%]	Right Ear (Total = 3) [50%]	Left Ear (Total = 3) [50%]	Right Ear (Total = 4) [57.14%]	Left Ear (Total = 3) [42.85%]
15–30 (Total = 3)	1[50%]	1[33.33%]	0	0	0	1[33.33%]
31–45 (Total = 9)	1[50%]	2[66.67%]	3[100%]	1[33.33%]	1[25%]	1[33.33%]
46–60 (Total = 6)	0	0	0	2[66.67%]	3[75%]	1[33.33%]

*P* Value ≤ 0.05 Significant

(Level of Significance *P* ≤ 0.05, Pearson Chi Square test)

## Conclusion

Involvement of the palatal muscles with OSMF may decrease the patency of the Eustachian tube, leading to conductive hearing loss. Therefore, as the test is non-invasive, painless and less time consuming, it can be effectively used for educating the patient, which may be helpful in assessing the morbidity and in identifying the overall prognosis to find more appropriate therapeutic interventions for hearing deficit in order to increase the success rate of treatment.
